# The Influence of Culture on Agroecosystem Structure: A Comparison of the Spatial Patterns of Homegardens of Different Ethnic Groups in Thailand and Vietnam

**DOI:** 10.1371/journal.pone.0146118

**Published:** 2016-01-11

**Authors:** Pijika Timsuksai, A. Terry Rambo

**Affiliations:** 1 Faculty of Agricultural Technology, Sakon Nakhon Rajabhat University, Sakon Nakhon, 47000, Thailand; 2 Program on System Approaches in Agriculture, Faculty of Agriculture, Khon Kaen University, Khon Kaen, 40002, Thailand; 3 The East-West Center, Honolulu, Hawaii, 96848–1601, United States of America; Institute of Botany, CHINA

## Abstract

Different ethnic groups have evolved distinctive cultural models which guide their interactions with the environment, including their agroecosystems. Although it is probable that variations in the structures of homegardens among separate ethnic groups reflect differences in the cultural models of the farmers, empirical support for this assumption is limited. In this paper the modal horizontal structural patterns of the homegardens of 8 ethnic groups in Northeast Thailand and Vietnam are described. Six of these groups (5 speaking Tai languages and 1 speaking Vietnamese) live in close proximity to each other in separate villages in Northeast Thailand, and 2 of the groups (one Tai-speaking and one Vietnamese-speaking) live in different parts of Vietnam. Detailed information on the horizontal structure of homegardens was collected from samples of households belonging to each group. Although each ethnic group has a somewhat distinctive modal structure, the groups cluster into 2 different types. The Tai speaking Cao Lan, Kalaeng, Lao, Nyaw, and Yoy make up Type I while both of the Vietnamese groups, along with the Tai speaking Phu Thai, belong to Type II. Type I gardens have predominantly organic shapes, indeterminate boundaries, polycentric planting patterns, and multi-species composition within planting areas. Type II homegardens have geometric shapes, sharp boundaries, lineal planting patterns, and mono-species composition of planting areas. That the homegardens of most of the Tai ethnic groups share a relatively similar horizontal structural pattern that is quite different from the pattern shared by both of the Vietnamese groups suggests that the spatial layout of homegardens is strongly influenced by their different cultural models.

## Introduction

A great deal of ethnoecological research has revealed that farmers belonging to different cultures have varying perceptions of the natural world, including the structure and functioning of their agricultural ecosystems [1]. Based on long-term trial and error experimentation by farmers, different ethnic groups have evolved distinctive cultural models of appropriate agroecosystem structures. These cultural models help guide their management decisions and interactions with the soil, water, plants and animals that make up their agroecosystems. Often these farmer models closely approximate the models developed by agricultural scientists. Sometimes the farmer models are superior to the scientific ones, but in other cases they are empirically deficient in varying ways [2]. Describing and understanding the cultural models of agroecosystems, including homegardens, of farmers belonging to different ethnic groups remains a major concern of ethnoecological investigations of agriculture, especially in developing countries in the tropics.

Homegardens occur on farmsteads in many parts of the tropical and temperate regions of the world. They are commonly, but not always, a relatively small subsidiary component of larger and more complex farm-level agroecosystems that may also include irrigated and/or dryland staple crop fields, pastures, and forest plots. Homegardens are most commonly used to produce food and other materials for household consumption (although they sometimes are also used to produce crops for the market, as in the case of the Viet ethnic group in this study).

Although it is highly probable that variances in the horizontal structure of homegardens among ethnic groups reflect differences in farmers’ cultural models, there is limited empirical evidence to support this assumption. Only a few systematic comparative studies of the homegardens of different ethnic groups have been published [1–9]. Moreover, the dimensions that are most commonly used to describe homegarden structure (surface area, vertical architecture, and species composition and diversity) [10–12] may not be reliable indicators of ethnic identity because they can be influenced by environmental and economic factors, rather than reflecting the traditional cultural models of the farmers. For example, the surface area of gardens is strongly influenced by population density and availability of land and gardens with small areas do not have enough space to grow tall trees. Species composition and diversity have also been shown to be influenced by both garden area and extent of commercial orientation [13]. The horizontal plans or layouts of gardens (e.g., shape of planting areas, definition of boundaries of plots within gardens, and planting patterns within plots), which are less subject to exogenous influences, and thus more likely to reflect the cultural models of the farmers, would seem to be more reliable markers of ethnicity. However, horizontal structure has received almost no attention in earlier research on homegardens anywhere in the world, and, in contrast to well-developed systems for describing vertical structure and species composition and diversity, there are no standard ways of describing horizontal structure.

In order to assess the extent to which different horizontal structural patterns of homegardens are associated with different ethnic groups, we carried out this comparative research on the homegardens of eight ethnic groups belonging to two different language families in Thailand and Vietnam. Six of these groups (5 belonging to the Tai language family and 1 belonging to the Vietnamese branch of the Mon Khmer language family) live in close proximity to each other in separate villages within the Sakon Nakhon Basin in Northeast Thailand and 2 of the groups (one Tai-speaking and one Vietnamese-speaking) live in different parts of Vietnam. We hypothesized that all culturally-related ethnic groups would have homegardens with broadly similar horizontal structural patterns, regardless of differences in their respective environments or exposure to neighboring groups with different garden structures. Thus, we anticipated that the homegardens of all of the Tai groups, regardless of whether they were in Northeast Thailand or in Vietnam, would have similar modal patterns and that the same would be the case for the Vietnamese groups. In this paper we present a system for classifying the key horizontal structural characteristics of homegardens, describe the modal horizontal structural characteristics of the homegardens of each of these ethnic groups, make a systematic comparative analysis of similarities and differences in the homegarden structures of the different ethnic groups, and relate these structural differences to differences in the general cultural patterns of the different groups.

## Methods

### Research approach

This study was designed to collect systematic data on the horizontal structure of homegardens of samples of households in rural communities representing the 8 ethnic groups included in this study. Because our preliminary observations revealed considerable variation in the structural characteristics of the homegardens of different households within the same ethnic community, we sought to analyze the data in such a way that would identify central tendencies without losing sight of the range of variation within each group. Therefore we employed a method devised by anthropologists to describe the modal personality structures of different cultures [14, 15]. Modal personality structure has been defined as “…the body of character traits that occur with the highest frequency in a culturally-bounded population. Modal personality is a statistical concept rather than the personality of an average person in a particular society” [16]. This approach is suitable for identification of central tendencies in populations that are internally heterogeneous. When applied to the study of homegardens, the goal is to identify those structural characteristics (e.g., organic or geometric form, lineal or polycentric planting patterns) that are found in the largest share of gardens of sample households belonging to each of the ethnic groups. Although our focus is on identification of modal tendencies, the frequencies with which alternative characteristics occur in each ethnic group sample are also shown.

### Selection of ethnic groups

**Fig 1:**
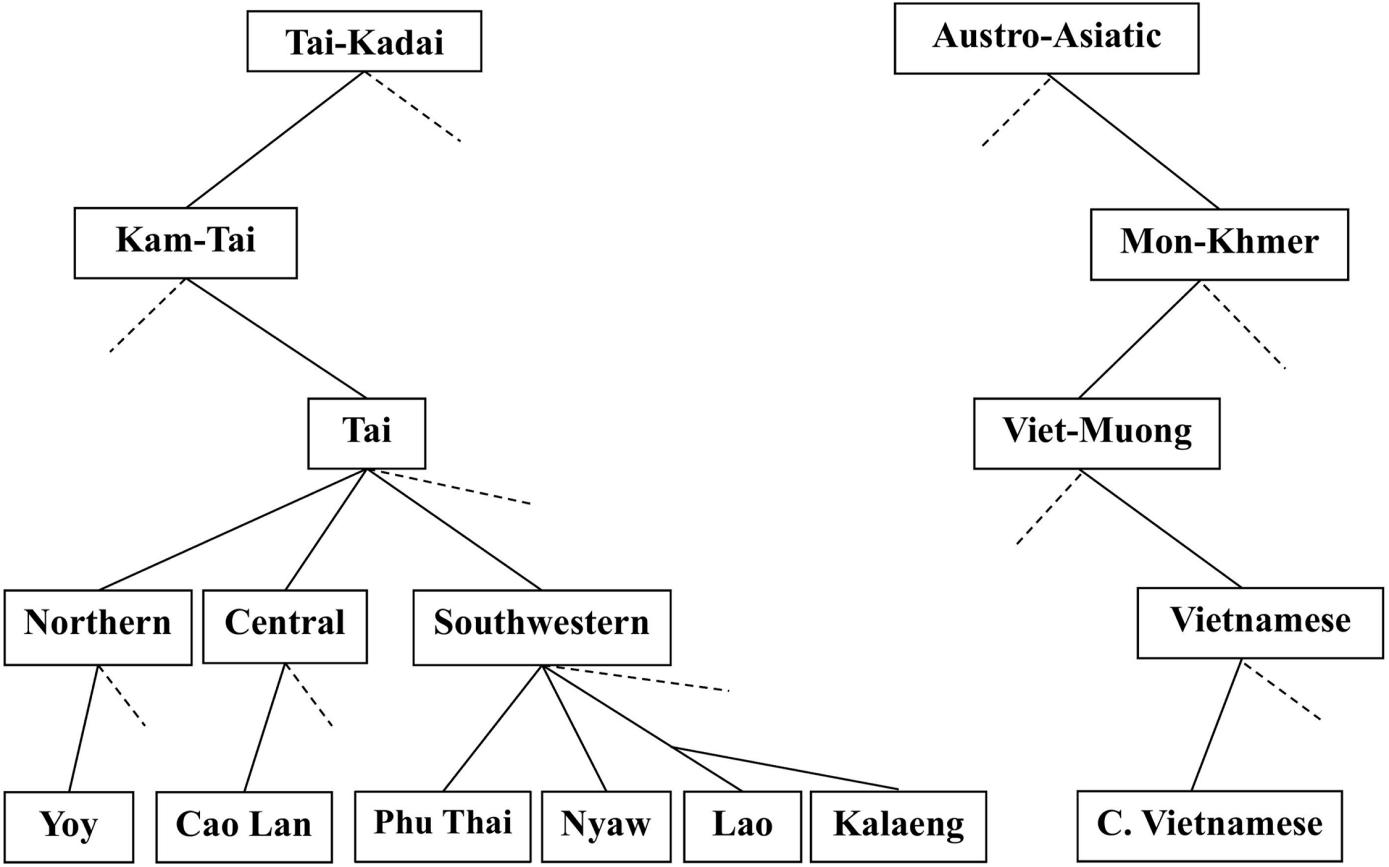
Ethnolinguistic taxonomy of groups in Northeast Thailand and Vietnam [44].

The northeastern region of Thailand is ethnically relatively homogeneous with members of the Thai Lao ethnolinguistic group (commonly referred to simply as “Lao”) forming the majority of the population [17]. However, the Sakon Nakhon Basin in the northern part of the region where we did this study has unusual ethnic diversity. The Lao, along with the Kalaeng, Nyaw, and Phu Thai, belong to the Southwestern group, the Yoy to the Northern group of the Tai language family, and the Viet (Thai Vietnamese) belong to the Vietnamese branch of the Mon-Khmer language family (Fig 1). The Cao Lan are a Tai speaking group in the Midlands of northern Vietnam who belong to the Central group of the Tai language family. They have had little or no contact with the Tai communities in Thailand for several hundred years. The Kinh (ethnic Vietnamese) in central Vietnam are the ancestral population of the Viet group in Northeast Thailand from whom they have been geographically isolated for more than a century.

There has been relatively little ethnographic research on most of the Tai groups. All of the Tai speaking groups are believed to have settled in the Sakon Nakhon Basin in the early nineteenth century after the Siamese army forcibly relocated them there from their homes in Laos [18]. Most of the Viet came to the area in the latter half of the nineteenth century, first fleeing the persecution of Catholic converts by the Vietnamese emperor Minh Mang and then escaping from the French colonial occupation of their homeland in central Vietnam. Later they were joined by refugees from the Indochina War in the late 1940s and after 1975 [19, 20]. The Cao Lan migrated into northern Vietnam from southern China several centuries ago [21, 22] and the Kinh (ethnic Vietnamese) are indigenous to central Vietnam.

### Selection of study sites

**Fig 2:**
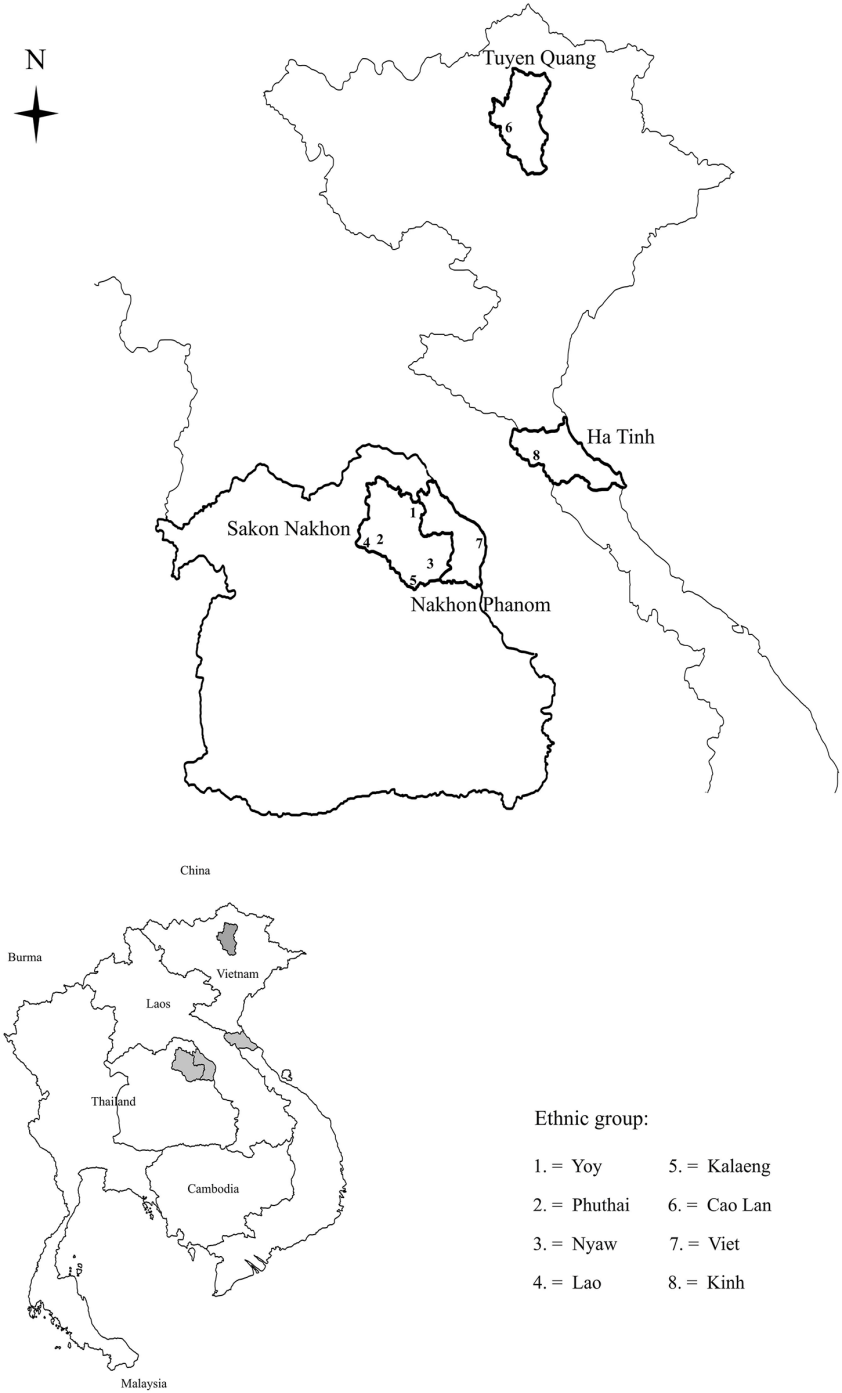
Map showing location of study villages in Northeast Thailand and Vietnam.

The study sites in Northeast Thailand were selected from rural villages representing the 5 Tai groups (Kalaeng, Lao, Nyaw, Phu Thai, and Yoy) and the Viet, all found within a relatively small area within the Sakon Nakhon Basin. In Vietnam, a Cao Lan village in a remote part of Tuyen Quang province was selected for study [23] along with a Kinh village in the district in Ha Tinh province from which the Viet living in Northeast Thailand had originally come. Knowledgeable local researchers and government officials were consulted in order to identify all of the villages inhabited by each ethnic group. The study villages were then selected on the basis of being located in a rural area, ethnically homogeneous, and having homegarden production mainly for household consumption. Semi-structured interviews were then conducted with village headmen and other villagers in order to confirm that the communities actually met the selection criteria. The locations of the study villages are shown in Fig 2. Table 1 presents information on the environmental and social characteristics of the study communities.

**Table 1:** Characteristics of study villages of different ethnic groups.

**Ethnic group**	Yoy	Phu Thai	Nyaw	Lao	Kalaeng	Cao Lan	Viet	Kinh
**Location (province, district, sub-district)**	Sakon Nakhon, Akat Umnuay, Akat Umnuay	Sakon Nakhon, Waritchaphum, Waritchaphum	Sakon Nakhon, Ponnakaew, Baan Paen	Sakon Nakhon, Song Dao, Tha Sila	Sakon Nakhon, Kud Bak, Kud Bak	Tuyen Quang, Son Duong, Dong Loi	Nakhon Panom, Muang Nakhon Panom, Nong Yat	Ha Tinh, Huong Khe, Huong Lien
**Geographic coordinates** ^1^	17° 36’00.83”N 103° 58’42.81” E	17° 16’52.06” N 103° 39’11.81” E	17° 11’41.83” N 104° 13’20.76” E	17° 14’38.03” N 103° 21’57.94” E	17° 04’09.34” N 103° 47’00.40” E	17° 22’12.80” N 104° 21’41.03” E	17° 22’38.09” N 104° 45’45.10” E	18° 03’46.04” N 105° 45’21.94” E
**Elevation (m amsl)** ^1^	152	193	166	214	212	169	156	83
**Topographical setting** ^2^	River bank	Hilly	Gently slopping	Hilly	Hilly	Mountain valley	Gently slopping	Mountain valley
**Land suitability**^3^	Loamy sand, infertile soil, good drainage	Loamy sand, infertile soil, good drainage,	Loamy sand, infertile soil, poor drainage	Sandy loam, infertile soil, moderately well drained	Loamy sand, infertile soil, good drainage,	Clay loam, infertile soil, well drained	Sandy loam or sandy clay loam, low to moderate infertile soil, poor drainage	Clay loam, infertile soil, well drained
**Area (ha)**^4^	50	488	760	536	800	120	202	40
**Population**^4^	510	1,058	556	655	788	76	520	376
**Population density (no. of people/km**^**2**^**)**	1,020	220	70	122	100	63	260	940
**No. of households**^4^	118	335	189	198	218	20	118	102
**Main purpose of homegardens**^5^	100% subsistence	55% subsistence, 45% commercial	100% subsistence	95% subsistence, 5% commercial	100% subsistence	100% subsistence	40% subsistence, 60% commercial	100% subsistence

Sources:

^1^GPS records of author;

^2^Observation by author;

^3^ Land Development Department (http://giswebldd.ldd.go.th/)(except for Cao Lan and Kinh groups from http://www.fao.org/ag/Agp/AGPC/doc/Counprof/vietnam/vietnam.htm);

^4^Village headman;

^5^ Samples of 20 homegardens(17 for the Cao Lan) in each village

### Selection of sample households in each community

Maps showing the location of all households in each village were drawn with the assistance of the village headman and/or village members who then drew a transect line across the center of the settlement area in order to provide a basis for sampling representative households. Starting from the first house at the beginning of the transect line, every house on both sides of the line that met our selection criteria was selected until a sample of 20 households (17 in the Cao Lan village) was achieved. For a household to be included in the sample, it had to meet the following criteria: 1) it had a homegarden, 2) its members belonged to the ethnic group under study, 3) it had been resident in the village for a minimum of two generations, and 4) an adult member granted us permission to observe and measure their homegarden. This work was done in accordance with the principles outlined in the Declaration of Helsinki. Although the Thai university agricultural faculties with which the authors are affiliated do not require human subjects review of non-medical research of this type, the research protocol was reviewed by the ethics board office of the Research and Development Institute of the first author’s university and classified as exempt due to low risk to human subjects. In the case of our study, no sensitive personal information was collected. Before we began data collection, the research was explained to the village head and his permission obtained to do the study in the village. At each of the sample households, the purpose of the research was explained to the farmers and their verbal permission obtained to observe and measure their gardens. It was explained that their participation was voluntary and they could opt out of the study at any time. All data in the paper are anonymous and cannot be traced to any particular individual informants. Although the sampling procedure does not meet the criteria of strict randomness, it did minimize the likelihood of unconscious bias on the part of the researchers influencing selection of sample households.

### Data collection and recording

Data were collected by means of direct observation and measurement of structural characteristics. Horizontal structure was recorded on sketch maps and by taking photographs. Data for the structural characteristics of all sample homegardens for each community were recorded in an Excel database which was used to compile comparative tables of garden structural characteristics for all of the study sites.

### Data analysis

**Fig 3:**
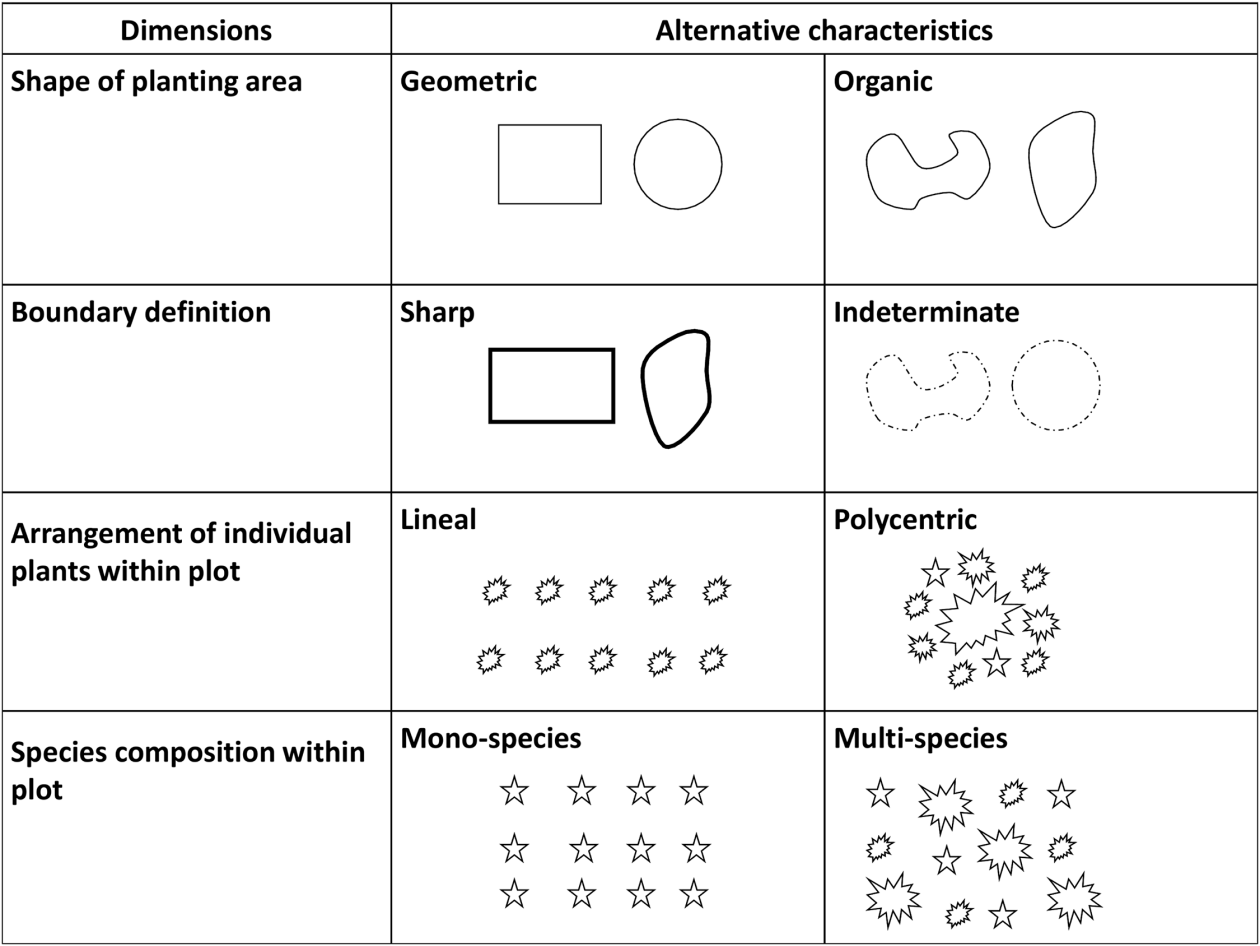
Classification system of horizontal structural characteristics of homegardens.

Because there are no standardized approaches for classifying horizontal structural dimensions of homegardens, we were compelled to develop our own analytic system. This system includes four different horizontal structural dimensions (Fig 3):

- Shape of planting areas or plots: *Geometric* forms include plots or beds with square, rectangular, or circular shapes. *Organic* forms include planting areas with irregular or curvilinear shapes.
- Definition of the boundary of the planting areas or plots: Boundaries can be *sharp* and clearly marked or *indeterminate* and ill-defined.
- Arrangement of individual plants within planting areas or beds: Individual plants can be planted in parallel lines (*lineal*) or in multiple clusters of plants (*polycentric*).
- Species composition within each plot: Planting areas or beds can be planted with only a single kind of plant species (*mono-species*) or with a mixture of two or more different species (*multi-species*).

Each homegarden of all of the sample households from each ethnic group was classified by a single researcher (the first author) in terms of the extent to which it manifested the alternative characteristics for each structural dimension. For example, the shapes of all of the planting areas within a garden were classified as being either geometric or organic and the surface area covered by each of these forms calculated. The garden was then categorized as to whether it was all geometric, >50% geometric, >50% organic, or all organic. The characteristic (e.g., all or mostly geometric) that was found to occupy more than 50% of the area in the largest number of gardens was selected as being modal for that structural dimension for that ethnic group. These data were then used to make a cluster analysis using the SPSS statistical package version 16.0 (SPSS Inc. Released 2007. SPSS for Windows, Chicago, SPSS).

## Results

Detailed information on the frequency of occurrence of different characteristics for each of the 4 horizontal structural dimensions for the sample of homegardens of each of the ethnic groups is presented in Table 2. Each of the ethnic groups has a single clearly dominant characteristic for each of the 4 structural dimensions (with the exception of the Yoy, for which equal shares [45%] of gardens have all mono-species and all multi-species planting patterns within beds). Table 3 presents the modal structural characteristics for each group.

**Table 2:** Comparison of horizontal structural characteristics of homegardens of different ethnic groups in Northeast Thailand and Vietnam (% of gardens displaying characteristic) (n = 20, except 17 for Cao Lan).

Structural dimension	Alternatives (%)	Tai groups						Vietnamese groups	
		Yoy	Phu Tai	Nyaw	Lao	Kalaeng	Cao Lan	Viet	Kinh
**Shape of planting areas**	**All Geometric**	**15**	**45**	**10**	**15**	**25**	**0**	**70**	**60**
	**>50% Geometric**	**0**	**20**	**5**	**5**	**0**	**0**	**15**	**25**
	**>50% Organic**	**15**	**15**	**30**	**5**	**0**	**28**	**0**	**15**
	**All Organic**	**70**	**20**	**55**	**75**	**75**	**72**	**15**	**0**
**Boundary definition of planting area**	**All Sharp**	**20**	**50**	**15**	**15**	**0**	**6**	**95**	**75**
	**>50% Sharp**	**5**	**25**	**20**	**5**	**40**	**0**	**0**	**10**
	**>50% Indeterminate**	**15**	**5**	**40**	**5**	**0**	**22**	**0**	**10**
	**All Indeterminate**	**60**	**20**	**25**	**75**	**60**	**72**	**5**	**5**
**Arrangement of individual plants within planting areas**	**All Lineal**	**15**	**5**	**15**	**15**	**25**	**11**	**75**	**55**
	**>50% Lineal**	**15**	**65**	**5**	**20**	**0**	**4**	**5**	**45**
	**>50% Polycentric**	**0**	**0**	**15**	**0**	**0**	**7**	**5**	**0**
	**All Polycentric**	**70**	**30**	**65**	**65**	**75**	**78**	**15**	**0**
**Species composition within planting area**	**All Mono-species**	**45**	**55**	**35**	**45**	**35**	**22**	**95**	**90**
	**>50% Mono-species**	**10**	**30**	**0**	**5**	**0**	**17**	**0**	**10**
	**>50% Multi-species**	**0**	**5**	**10**	**0**	**0**	**0**	**0**	**0**
	**All Multi-species**	**45**	**10**	**55**	**50**	**65**	**61**	**5**	**0**

**Table 3:** Comparison of modal structural characteristics of homegardens of different ethnic groups in Northeast Thailand and Vietnam (% of homegardens with all or >50% of their area displaying each characteristic) (n = 20, except 17 for Cao Lan).

Dimension	Tai groups						Vietnamese groups	
	Yoy	Phu Thai	Nyaw	Lao	Kalaeng	Cao Lan	Viet	Kinh
**Shape of planting area**	Organic (85%)	Geometric (65%)	Organic (85%)	Organic (80%)	Organic (75%)	Organic (100%)	Geometric (85%)	Geometric (85%)
**Boundary definition of planting areas**	Indeterminate (75%)	Sharp (75%)	Indeterminate (65%)	Indeterminate (80%)	Indeterminate (60%)	Indeterminate (94%)	Sharp (95%)	Sharp (85%)
**Arrangement of individual plants within planting area**	Polycentric (70%)	Lineal (70%)	Polycentric (80%)	Polycentric (65%)	Polycentric (75%)	Polycentric (85%)	Lineal (80%)	Lineal (100%)
**Species composition within planting area**	Mono-species (55%)	Mono-species (85%)	Multi-species (65%)	Mono-species (50%) Multi-species (50%)	Multi-species (65%)	Multi-species (61%)	Mono-species (95%)	Mono-species (100%)

Organic shaped planting areas, indeterminate boundaries, and polycentric planting patterns are modal for the Cao Lan, Kalaeng, Lao, Nyaw, and Yoy, while for the Phu Thai, Kinh, and Viet geometric forms with sharp boundaries and lineal planting patterns are modal (although a sizable minority of Phu Thai gardens have organic or mostly organic shapes, indeterminate or mostly indeterminate borders, and polycentric planting patterns). Planting of multiple species in the same planting area is modal for the Cao Lan, Kalaeng, and Nyaw, and while the Phu Thai, Kinh, and Viet have mono-species planting areas and the Yoy and Lao have equal shares of gardens with mono- and multi-species beds.

**Fig 4:**
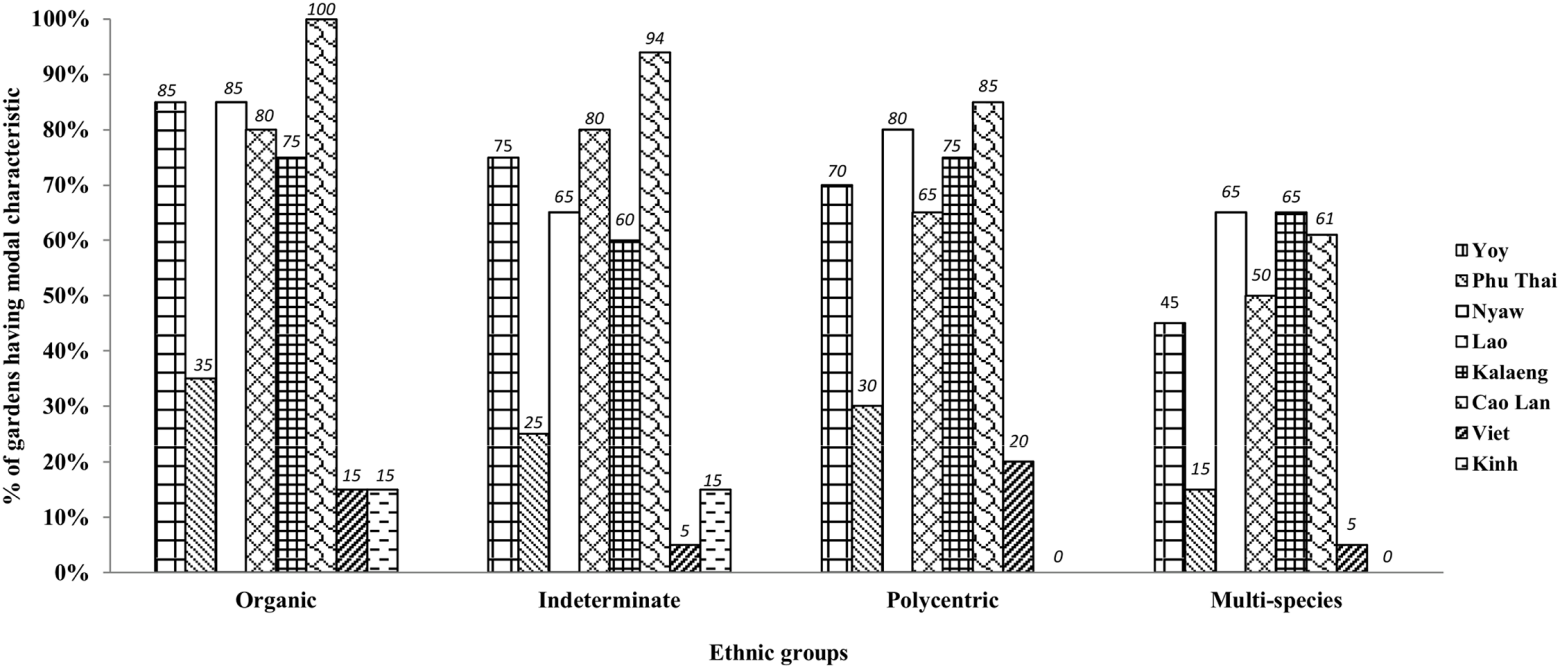
Comparison of modal structural patterns of homegardens of different ethnic groups in Northeast Thailand and Vietnam (% of gardens having characteristic).

Fig 4 is a graphic comparison of the modal patterns of each of the groups. The patterns of all Tai groups, with the exception of the Phu Thai, are quite similar to one another, although the Cao Lan pattern is the most distinct and does not fully overlap with the other Tai patterns. The Kinh and the Viet patterns are almost identical while the Phu Tai pattern is closer to that of the Vietnamese groups than it is to the other Tai groups.

**Fig 5:**
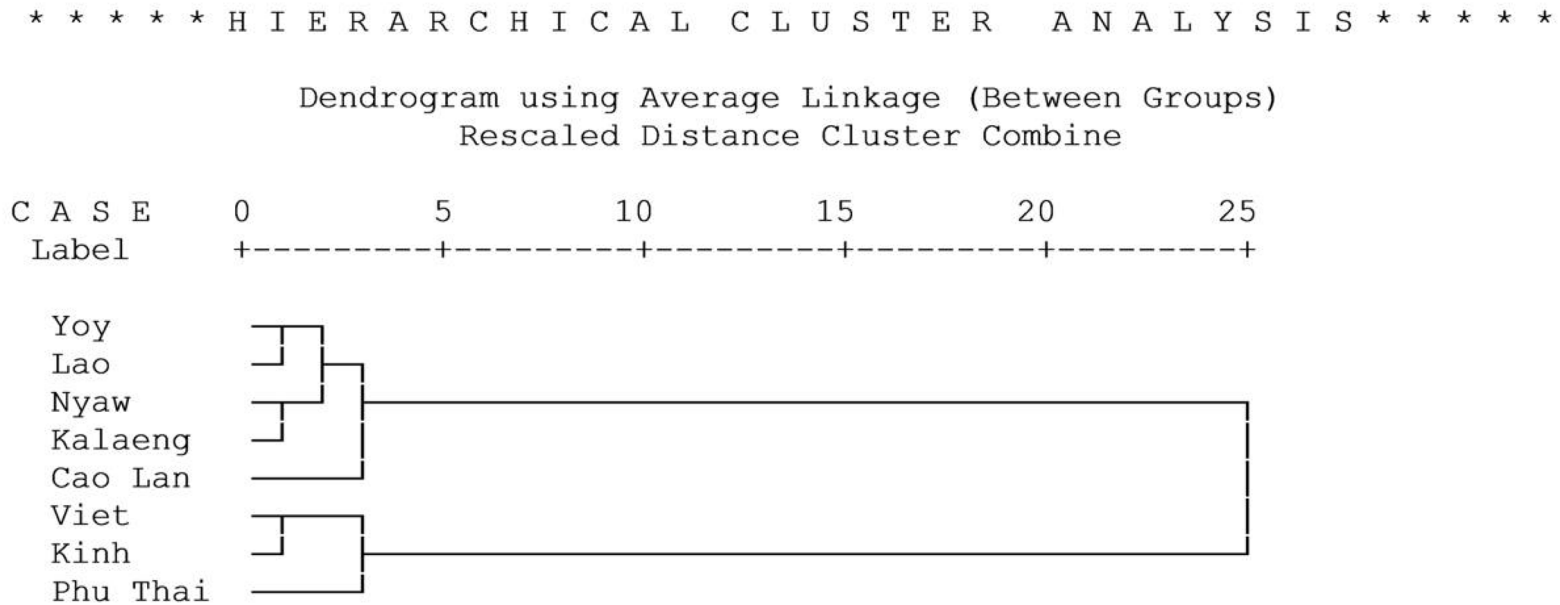
Hierarchical cluster analysis based on percentages of modal characteristics of structural dimensions of homegardens of ethnic groups in Northeast Thailand and Vietnam.

Fig 5 presents a hierarchical cluster analysis of the modal structural characteristics of the homegardens of the 8 ethnic groups. They cluster into two main types: Type I (Cao Lan, Kalaeng, Lao, Nyaw, and Yoy) and Type II (Phu Thai, Kinh and Viet). Within Type I, the Cao Lan are a separate sub-type while the Phu Thai are a separate subtype within Type II. Homegardens of Type I are characterized by having predominantly organic shapes, indeterminate boundaries of planting areas, polycentric planting patterns, and multi-species composition within planting areas. Homegardens of Type II have geometric shapes, sharp boundaries, lineal planting patterns, and mono-species composition of planting areas. However, the Phu Thai homegardens, although they belong to Type II, are less homogenous than those of the Vietnamese groups and show resemblance to Type I in some regards. Thus, although geometric shapes, sharp boundaries, lineal planting patterns, and mono-species composition are modal, organic shapes, indeterminate boundaries of planting areas, and polycentric planting patterns are also encountered in a considerable minority of their gardens.

## Discussion

There is a strong association between ethnicity and the horizontal structure of homegardens. The homegardens of most of the Tai ethnic groups share a relatively similar horizontal structural pattern, one that is quite different than the pattern shared by both of the Vietnamese groups. Although we are well aware of the axiom that correlation does not equal causation, we believe that it is legitimate in this case to infer that the spatial layout of homegardens is strongly influenced by the cultural models of the different ethnic groups and not the reverse. The Tai groups in Northeastern Thailand have had no direct contact with the Cao Lan in Vietnam for many centuries and yet their gardens display very similar horizontal structural patterns. At the same time, the structural pattern of the homegardens of the Viet in Northeastern Thailand is virtually identical to that of the Kinh in Vietnam from whom they have been separated for more than 100 years. The persistence of this common pattern, despite the fact that the Viet have lived in close proximity with neighboring Tai groups in Northeastern Thailand for several generations, is remarkable since there should have been sufficient time for convergence on a common modal pattern to have occurred if environmental selective forces and/or acculturative pressures were the main determinants of agroecosystem structure. Studies of the homegardens of immigrant minority groups in other parts of the world have also found that they commonly replicate the garden patterns of their homelands rather than adopting the pattern of the majority populations of the countries where they have resettled. For example, the vegetable gardens of Vietnamese refugees living in New Orleans in the United States have similar planting patterns and species composition to homegardens in Vietnam [24]. The widespread persistence of distinctive agricultural patterns in immigrant communities in new environmental circumstances [25–27] provides further evidence that culture is an important determinant of agroecosystem structure.

Although not amenable to quantitative analysis, it appears that the structural patterns of the homegardens of the Tai ethnic groups are highly congruent with the other Tai cultural patterns while the structural patterns of the Vietnamese gardens are congruent with broader Vietnamese cultural patterns. In particular, we would suggest that differences between the Tai and Vietnamese gardens in the spatial arrangement of plants within the gardens and the extent to which planting areas are clearly delineated reflect important differences in basic Tai and Vietnamese cultural patterns. The Tai gardens, which are polycentric and mix together many different species in the same organically shaped planting areas, may seem to an outside observer to be unplanned and lacking in order in comparison to the straight rows of plants of a single species in the neatly laid out geometric beds of the Vietnamese gardens. The same seeming lack of order has often been noted as a general characteristic of Thailand’s society, which was famously characterized by John Embree [28] as being “loosely structured.” Embree, an American anthropologist who had done extended ethnographic research in Japan before coming to Thailand, was struck by the seeming lack of order in Thai social life in comparison to the highly codified patterns of Japanese society. Of course, although Embree failed to perceive it, there is an underlying order in Thai society [29], but it is of a very different nature than the more rigidly defined social order in Chinese-influenced cultures such as Japan and Vietnam [30]. Differences in the sharpness with which the boundaries of planting areas are defined in Tai and Vietnamese gardens may also reflect more general cultural patterns of these societies. In comparison to the sharp borders of Vietnamese garden beds, the planting areas of the Tai gardens lack clearly demarcated edges or borders. This is congruent with a more general lack of concern in Tai culture with demarcating territorial boundaries. It was only in the mid- nineteenth century when, under pressure from the British and French, the Kingdom of Siam first began to map its territorial borders [31]. Only in the 1960s, encouraged by government rural development workers, did Northeastern Thai villagers begin to build fences to mark the borders of their house plots [32]. In contrast Vietnamese culture strongly emphasizes the delineation of clear boundaries, including of the borders of the national territory, of individual villages, which were traditionally surrounded by a bamboo hedge or earthen wall and of individual house plots within villages [33].

The finding that the homegardens of the Phu Thai have a structural pattern that is closer to the Vietnamese pattern than that of the other Tai groups does not fit with our initial hypothesis and is difficult to explain using the very limited available historical and ethnographic information about the Tai ethnic groups in Northeast Thailand. However, the Phu Thai are commonly recognized as being culturally quite distinct from other Tai groups. After they were resettled in Thailand in the 1800s, they lived in a largely autonomous ethnic enclave with their own ruler and had very limited contact with other Tai groups in the area. At present they have a reputation among other Tai for being hard-working and innovative. Their economy is more productive, and they have been very quick to diversify their agriculture into production of a variety of cash crops [34]. We observed that their village was better organized and exhibited greater social cohesion than the other Tai communities included in our study.

The continuing coexistence within the same geographical area of homegardens with two quite different structural patterns raises questions about the extent to which agroecosystem structure is determined by environmental factors as is often assumed to be the case [35, 36]. The Type I homegardens of the Tai groups resemble the tropical forest model of homegardens first proposed by Terra [3–5] and later elaborated by Soemarwoto [37] and researchers associated with the International Center for Research in Agroforestry (ICRAF) (e.g., 10–12). In gardens of this type, the planting pattern has been variously characterized as having uneven or random spacing, or even as being in “disarray,” with individual plants of different species scattered at seemingly random intervals within the garden area [38]. The structure of Type II gardens of the Phu Thai and both of the Vietnamese groups resembles the “temperate type” homegardens described by Niñez [39]. Temperate type gardens are characterized by neatly arranged plantings of mostly annual crops of uniform height in mono-specific rectangular beds. Tropical type homegardens are indigenous to Southeast Asia [3–5] while the temperate type of homegardens probably originated in China [40]. The latter type subsequently spread to Southeast Asia, first to Vietnam, while it was under Chinese political domination, and subsequently, during the European colonial era to Malaya and other colonies where it was introduced by Chinese migrants [41]. The fact that temperate type homegardens function successfully in both Northeast Thailand and central Vietnam, which have tropical climates, suggests that environmental selection is not very rigorous and that both types are essentially equally well-adapted to tropical conditions.

## Conclusions

Study findings suggest a close linkage between ethnicity and the structure of homegarden agroecosystems. Most of the Tai groups share a common structural pattern for their homegardens while both of the Vietnamese groups share their own common structural pattern. This close association between ethnicity and agroecosystem structure represents what Richard O’Conner [42], in his study of ethnic competition in the history of Southeast Asia, has referred to as an “agro-cultural complex.” These complexes have persisted through time and space and retained their integrity, even when the ethnic groups on which they are based have migrated into different environments and encountered strong acculturative pressures from neighboring populations having different ethnic identities and distinctive agroecosystem models.

The existence of such strong and durable links between ethnic identity and agroecosystem structure has important implications for research on agricultural development. Agricultural research has been heavily dominated by economic and technological concerns, reflecting the assumption of agricultural scientists and government policymakers that farmers, regardless of their ethnic identity, will always tend to adopt agricultural structures and practices that provide optimum economic returns [27]. To the extent, however, that agroecosystem structures reflect the cultural models of the farmers, adoption of improved technology may be constrained by its compatibility with these models. It is possible, of course, that homegardens, which are mostly small plots used to meet household subsistence needs, are more likely to conserve traditional cultural patterns because they are less subject to market pressures to maximize productivity than cash-cropping components of agroecosystems. However, this is not necessarily the case since we know that even modern American commercial farmers are influenced by cultural factors, as shown, for example, by their initial resistance to adoption of economically beneficial sustainable agriculture partly because this system was associated in the popular imagination with “hippies” [43]. Therefore, assessing the ways in which the cultural beliefs and values of farmers from different ethnic groups influence their choice of appropriate agricultural structures and practices should have an important place on the research agenda of agricultural researchers and policymakers in developing countries.
